# Uncovering Oncogenic Mechanisms of Tumor Suppressor Genes in Breast Cancer Multi-Omics Data

**DOI:** 10.3390/ijms23179624

**Published:** 2022-08-25

**Authors:** Seong Beom Cho

**Affiliations:** Department of Biomedical Informatics, College of Medicine, Gachon University, Incheon 21565, Korea; sbcho1749@gachon.ac.kr

**Keywords:** tumor suppressor gene, multi-omics data, breast cancer, regulatory mechanism

## Abstract

Tumor suppressor genes (TSGs) are essential genes in the development of cancer. While they have many roles in normal cells, mutation and dysregulation of the TSGs result in aberrant molecular processes in cancer cells. Therefore, understanding TSGs and their roles in the oncogenic process is crucial for prevention and treatment of cancer. In this research, multi-omics breast cancer data were used to identify molecular mechanisms of TSGs in breast cancer. Differentially expressed genes and differentially coexpressed genes were identified in four large-scale transcriptomics data from public repositories and multi-omics data analyses of copy number, methylation and gene expression were performed. The results of the analyses were integrated using enrichment analysis and meta-analysis of a *p*-value summation method. The integrative analysis revealed that TSGs have a significant relationship with genes of gene ontology terms that are related to cell cycle, genome stability, RNA processing and metastasis, indicating the regulatory mechanisms of TSGs on cancer cells. The analysis frame and research results will provide valuable information for the further identification of TSGs in different types of cancers.

## 1. Introduction

Tumor suppressor genes (TSGs) are a functional category of genes involved in normal cellular processes; however, the loss of their functions results in cancer development and progression [1,2]. In normal cells, TSGs have a role in cell cycle regulation [3], DNA damage repair [4], ubiquitination [5], and cellular differentiation [6]. Genetic mutations cause TSGs’ aberrant functions [7]. For example, p53 is a representative TSG that shows gain-of-function activities in cancer including augmenting cancer cell survival [8], altering the metabolism of cancer cells to adapt to the changing microenvironment of cancer [9], and preventing proteolysis responding to oncogenic transformation of cells [10] when the gene product is mutated. The phosphatase and tensin homolog (PTEN) is another well-known TSG with tumor-suppressive activities through cell cycle inhibition and induction of apoptosis [11,12].

The TSGs present functional roles through regulatory interaction with other genes [1,13]. The inhibition of cell-cycle machinery is accomplished by the formation of E2F and retinoblastoma protein (RB), which is a well-known TSG [3]. The adenomatous polyposis coli (APC) gene is a tumor suppressor with a negative regulatory effect on Wnt signaling, which induces apoptosis [14]. The mutS homolog 2 (MSH2) loses its tumor-suppressive function via abnormal mismatch repair system [15]. TSGs such as TIMP-3 are associated not only with tumorigenesis, but also with cancer cell metastasis [16]. Therefore, their therapeutic possibilities have been studied [17]. The findings indicate that TSGs are involved in cancer pathophysiology and the identification of a gene regulatory relationship between TSGs and the other genes is essential in developing new therapeutic strategies for cancers.

**Figure 1:**
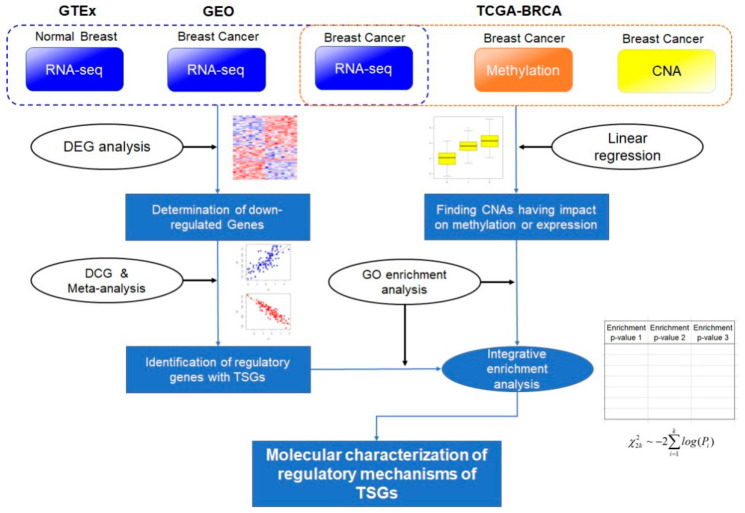
Schematic diagram of the whole analysis process. In this analysis, four RNA sequencing datasets, whole genome-wide copy number alterations (CNAs), and methylation data were used. RNA sequencing (RNA-seq) data of normal breast and breast cancer cells were collected from the Gene Expression Omnibus (GEO) database and Genotype-Tissue Expression (GTEx) project. The multi-omics data containing the CNA, methylation, and RNA-seq data of breast cancer cells were obtained from The Cancer Genome Atlas (TCGA) project. Using the RNA-seq data, differentially expressed gene (DEG) and differential coexpression (DCE) analyses were performed. In the DCE analysis, results were integrated using a meta-analysis of *p*-value summation (see Materials and Methods). Breast cancer multi-omics data from the TCGA project were used in the determination of CNAs having effects on methylation or gene expression. For each tumor suppressor gene (TSG), significant results of the DCE, CNA methylation, and CNA expression analyses were used as input for gene ontology (GO) enrichment analysis. The results were integrated using a *p*-value summation method. This process identifies the GO biological process terms that are related to the regulatory mechanisms of TSGs.

The discovery of TSGs was enabled by analyzing the results of genetic and molecular biologic experiments. Sequence-based information from mutations and structural variations were used in most studies [18,19,20,21,22,23]. However, the emergence of functional genomics data allows the TSGs and their regulatory actions to be screened more efficiently [24,25,26]. Following the advent of multi-omics data, such as The Cancer Genome Atlas (TCGA) datasets [27], it has become more convenient to find TSGs in a variety of cancers [28,29]. In this study, a meta-analysis of transcriptomics data was performed to identify the regulatory relationships between TSGs and other genes involved in cancer pathophysiology. Moreover, multi-omics data were used to select TSGs. All data were obtained from public databases with open genomics data. Figure 1 shows a graphical abstract of the analysis flow in this research.

## 2. Results

### 2.1. Data Preprocessing and Differentially Expressed Gene Analysis

In this analysis, four sets of RNA sequencing (RNA-seq) data, including normal breast data from the Genotype-Tissue Expression (GTEx) project (54,592 × 459), breast cancer data from the TCGA project (58,037 × 1246), GSE96058 (30,865 × 3409), and GSE81538 (17,641 × 405), were used. The numbers in parentheses indicate the number of genes and samples, respectively. In total, the gene expressions from 5519 samples were used in the analysis. Gene symbol identifiers were used for transcript annotation. The RNA-seq data had different scales of expression values, including counts (GTEx project data) and *z*-transformed values of the counts (TCGA, GSE96058 and GSE81538). For determination of differentially expressed genes (DEGs), the gene expression scale should be adjusted for fair comparisons of gene expression between normal breast and breast cancer expression profiles. The GSE96058 and GSE81538 data provided the normalized expression values only. Thus, normalization methods, such as the fragments per kilobase of exon per million (FPKM) method, could not be applied. Moreover, the data might have batch effects that hinder DEG analysis [30]. To eliminate the difference in the scales of expression values and possible batch effects, the expression values were transformed into gene expression ranks, which indicate that the ranks of expression values were compared between groups instead of the expression values. Because the batch effect causes a shift in all gene expression patterns in a sample, the ranks of gene expression values in a sample are maintained even if the batch effect changes the gene expression values. Because the gene expressions were transformed into ranks, we can compare the degrees of gene expression without considering the different scales. Moreover, the ranks were determined sample by sample; therefore, the ranks were tolerant to the batch effect, which affects all genes of a sample equally. Thus, if the ranks of the gene expression values were used as surrogates for real expression counts, the attenuation of the batch effect could be expected.

To remove the difference in scales of expression values and possible batch effects, expression values were transformed into gene expression ranks, which indicates that ranks of expression values were compared between groups, instead of the expression value itself. First, the common genes included in the normal and breast cancer RNA-seq data were determined and their expression values extracted. In each sample, gene expression values were then sorted in ascending order and the corresponding ranks were substituted in the expression values. The DEG analysis with the Wilcoxon rank-sum test was performed on the rank-transformed expression values from the normal and breast cancer data. The four RNA-seq datasets included 16,876 common genes, and 459 normal breast and 5060 breast cancer samples. The Wilcoxon rank-sum test identified 14,847 significant DEGs with Bonferroni’s multiple testing correction (*p*-value < 2.96 × 10^−6^ = 0.05/16,876). The large number of DEGs might result from following reasons. First, the enlarged number of samples might increase the power of the DEG analysis. Second, the number of genes that were restricted to the common genes from the four datasets, which resulted in reducing the number of tests. This had the effect of relaxing the *p*-value threshold, while otherwise being more stringent, with an increased number of genes. The relaxed *p*-value threshold seemed to increase the number of the significant genes in the DEG analysis.

**Figure 2:**
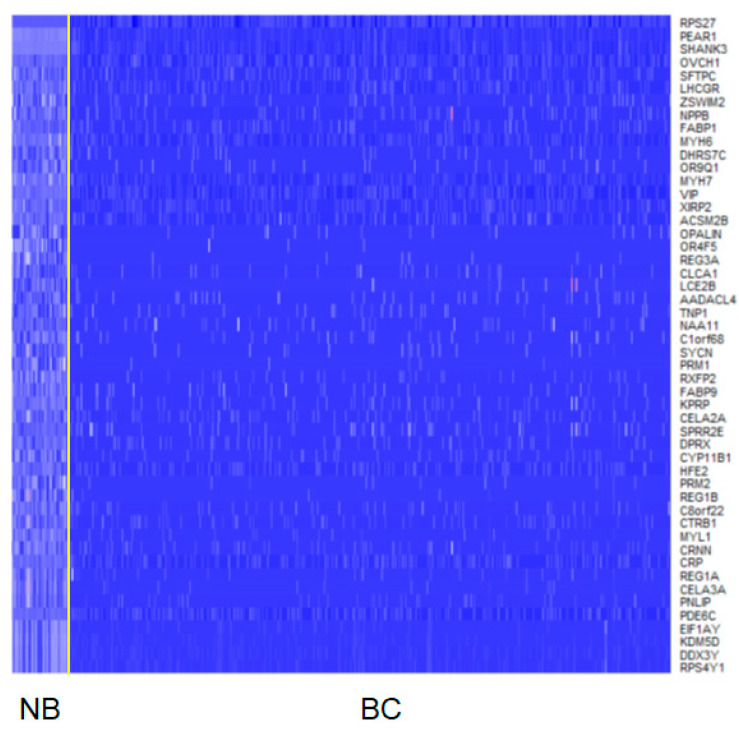
Heatmap of top 50 downregulated genes. The heatmap is drawn with ranks of gene expressions. The yellow line discriminates samples from normal breast and breast cancer. NB, normal breast; BC, breast cancer.

In the determination of tentative TSGs, downregulated DEGs were used because TSGs are genes that show losses of their normal functions in cancer tissues. A ratio of median rank of expressions of a gene in breast cancer to that in normal breast was used for the determination of TSGs. The downregulation was assigned when the ratio was below 1. Of the 14,847 DEGs, 7318 genes showed gene expression downregulation (Figure 2 and Supplementary Table S1). Among the genes, 508 TSGs had been determined previously using genetic and epigenetic features [31]. The remaining 6810 genes were treated as non-TSGs. Of the TSGs, RAPGEF3 showed the most significant result (*p*-value = 1.13 × 10^−275^, Supplementary Table S1). The RAPGEF3 is also known as EPAC1, which supports metastasis-related biological processes (BPs), such as angiogenesis, cell migration, and invasion in triple negative breast cancer cell lines [32]. BCL6 was the second most significant in the results and it is associated with survival of breast cancer cells [33]. The ratios for the median rank of gene expressions in breast cancer to those in normal breast were much higher in the TSGs. If the threshold of the ratio was set to 0.5, which means the median rank of a gene expression in breast cancer is doubled in normal breast, eight TSGs were included. In the remaining 6810 downregulated genes; however, the ratios of 705 genes were less than 0.5. When the distribution of such ratios in TSGs and non-TSGs of the downregulated genes was tested using Fisher’s exact test, the results indicated that genes with higher degrees of downregulation are enriched in non-TSGs (odds ratio = 0.14, *p*-value = 2.36 × 10^−14^). These results are consistent with an earlier study [34].

### 2.2. Differential Coexpression Reveals Genetic Regulatory Network of Tsgs in Breast Cancer

For identification of differentially coexpressed gene pairs (DCGs), the coexpressions between genes were determined using Pearson’s correlation coefficient (PCC) in each dataset. The significant differences in the coexpressions between normal and cancer tissues were tested statistically using the standard normal distribution (see Materials and Methods). In this study, the 7318 downregulated DEGs were used for the differential coexpression analysis to focus on revealing the regulatory network for TSGs and other downregulated genes. While DEG analysis used rank-transformed values to avoid possible batch effects, the original expression values of the four RNA-seq data were used in the DCG analysis because the comparisons of coexpressions are performed with correlation coefficients, not the expression values directly. GSE96058 and GSE81538 provided normalized expression values that were used for DCG analysis. The GTEx and TCGA project data included gene expression counts, and log transformation was applied to the data to shape the distribution closer to a normal distribution. For log transformation of gene expressions with zero count, a constant (=1) was added to all the count values from the two datasets.

We applied a hard thresholding approach in the estimation of significant DCGs, where only the significant coexpressions in normal breast and breast cancer were included in the DCG analysis. In addition, gene pairs with absolute coexpression (absolute PCC) values of over 0.3 were included for greater confidence in the coexpression gene pairs from normal breast tissues in the analysis. In the determination of significant results, Bonferroni’s multiple testing correction was applied. The adjusted *p*-value was 1.87 × 10^−9^ (=0.05/26,772,903), where the denominator was the number of possible gene pairs of the 7318 downregulated genes. After all pairwise coexpressions had been determined independently, only the concurrently significant pairs in the four datasets were included in the subsequent analysis. In the DCG analysis, three DCG *p*-value vectors were generated (normal versus GSE96058, GSE81538, and TCGA breast cancer data, respectively) and integrated. The *p*-value vectors were likely to have correlations because the same normal breast data were used for the different DCG analyses. Such correlations tend to inflate the statistics obtained by the Fisher *p*-value summation method, which assumes independent *p*-values in its original form. Therefore, the empirical Brown method was used for meta-analyses instead of Fisher’s *p*-value summation method. This method was originally developed to integrate a correlation structure between genes in a gene set analysis [35]. In this analysis; however, the method was used for integrating correlations between the three *p*-value vectors.

**Figure 3:**
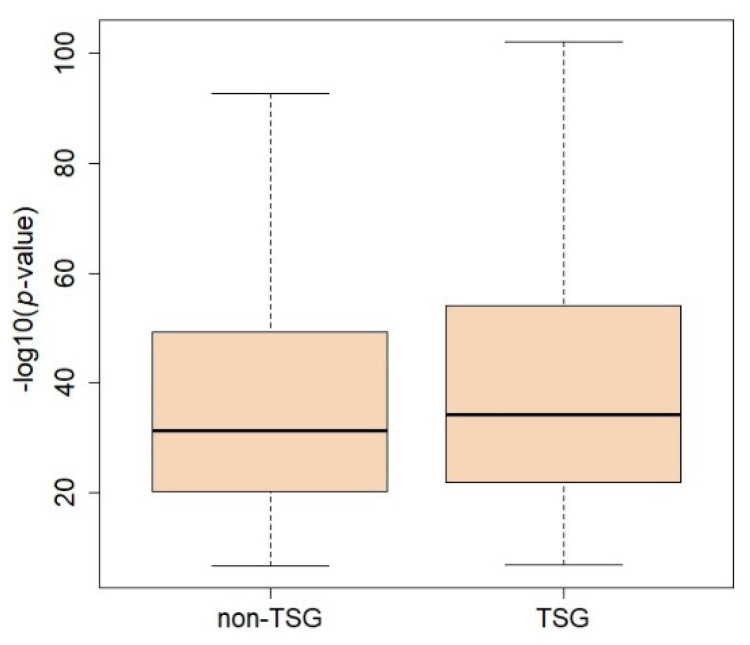
Comparison between *p*-values from differential coexpression (DCG) analysis. The tumor suppressor gene (TSG) group contains DCG results of gene pairs that have at least one TSG in the pair.

**Figure 4:**
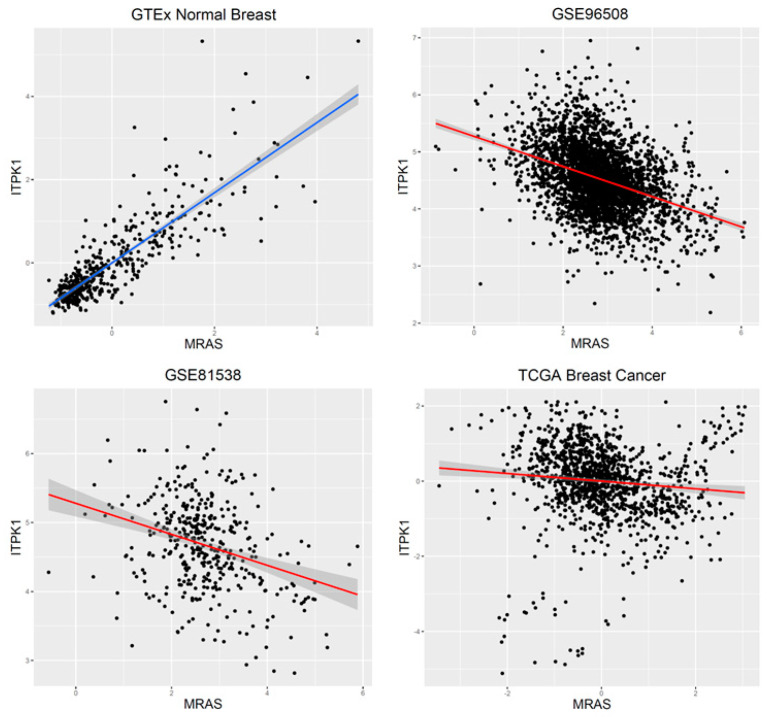
Differential coexpressions between MRAS and ITPK1 genes. Please note that MRAS and ITPK1 show a highly positive correlation in normal breast, while the show a negative correlation in the breast cancer data. GTEx, Genotype Tissue Expression project; TCGA, The Cancer Genome Atlas project.

In total, there were 1,385,777 DCGs found to be significant with the Empirical Brown method (Bonferroni’s adjusted *p*-value = 3.60 × 10^–8^, Table 1 and Supplementary Table S2). As expected, Fisher’s *p*-value summation method produced more significant p-values, which indicates the inflation of statistics (Supplementary Table S2). An interesting coexpression pattern was found in the set of significant results. While the absolute values of coexpressions in normal breast were used, all the coexpressions from the normal breast data were positive, whereas those from the breast cancer data were negative in all datasets. A total of 190,678 gene pairs had at least one TSG, and the difference in *p*-values between pairs with TSGs and with no TSGs was highly significant (Figure 3, Wilcox rank-sum test *p*-value < 2.2 × 10^–16^). The MRAS and ITPK1 gene pair showed the most significant results (Figure 4). In the normal breast data, they showed a strong coexpression (0.84), and the coexpression was reversed to negative coexpressions in the breast cancer data (i.e., −0.39, −0.31, and −0.10 in the GSE96058, GSE81538, and TCGA data, respectively).

**Figure 5:**
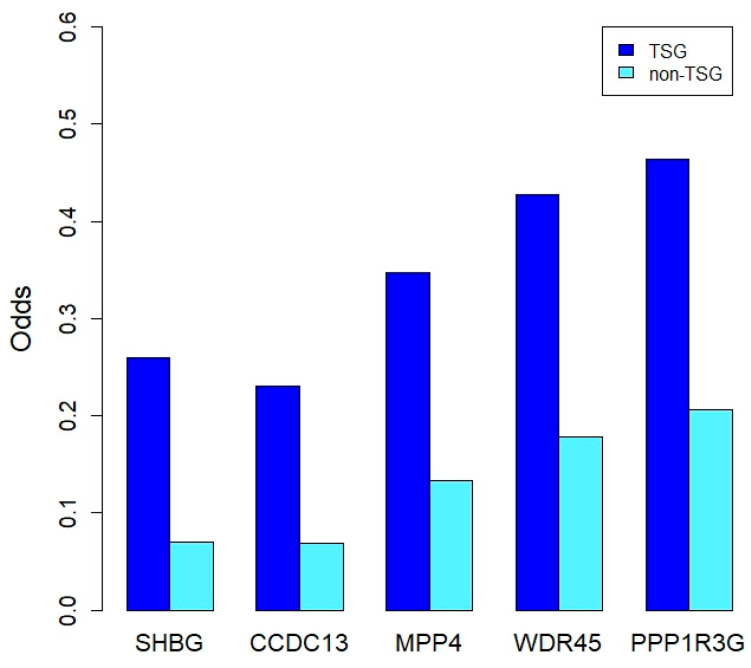
Genes that have differential coexpressions with TSGs more frequently. The y axis indicates odds that are a ratio of the number of differentially co-expressed genes with tumor suppressor genes to the number of no co-expressed genes for a single gene. Therefore, a high odds ratio indicates that a gene is more frequently significant with TSGs, implying that the gene has regulatory relationships with TSGs.

In the results of the DCG analysis, each of the 7318 downregulated genes was differentially coexpressed with 379 genes, on average. Some genes showed different coexpression with TSGs more frequently than with non-TSGs. Fisher’s exact test was applied to reveal this tendency in the DCGs of the downregulated genes (Supplementary Methods). Here, the differential coexpression represents significant changes in coexpression measured using the correlation coefficients of two genes between normal breast and breast cancer cells. Therefore, the differential coexpression represents the changes in the regulatory relationships of gene pairs related to malignant transformations in breast cancer. In addition, if a gene has more differential coexpression relationships with TSGs than non-TSGs, it is possible that the gene is more important in the regulation of TSGs, because the significance of the high odds ratio indicates that the result is not a random event. The significant genes identified in the test might be the core regulators of TSGs in breast cancer. After Bonferroni’s multiple testing correction (adjusted *p*-value threshold = 6.83 × 10^−6^), 179 genes were found to have more DCG links in TSGs. The sex-hormone-binding globulin (SHBG) gene was the most significantly enriched in TSGs (Figure 5, Supplementary Table S3). To characterize the genes, functional annotation analysis was applied with the GO BP terms and KEGG pathway gene sets. The 7318 downregulated genes were set to background genes of the enrichment analysis. In the results of the gene ontology annotation, the ‘cytoplasmic translation’ (GO:0002181, *p*-value = 3.47 × 10^–8^) biological process term was significant with multiple testing correction. When the Kyoto Encyclopedia of Genes and Genomes (KEGG) pathway information was used, the ‘Ribosome’ (hsa03010, *p*-value = 2.20 × 10^–12^) pathway was significant. These results showed that ribosome-related BPs are important in the regulation of the TSGs of breast cancer.

### 2.3. Identification of Copy Number Alterations Having an Impact on Methylations and Gene Expressions

Copy number alterations (CNAs) indicate a change in the genome structure related to TSGs [29]. Therefore, if CNAs have functional impacts on gene expressions or other functional changes, they have a high possibility of being a TSG. For the identification of such functional impacts of CNAs, multi-omics data analyses were performed with CNAs, methylation, and gene expression data from the TCGA breast cancer dataset downloaded from the cBioPortal website [36].

As described in the Materials and Methods, a linear model was applied to detect CNAs with an impact on methylation or gene expression. In the analysis, methylations or gene expressions were regressed on CNAs of the corresponding genes. When methylations and gene expressions were fitted to CNAs, only the cis effect of CNAs of the corresponding gene was tested. In the CNA-methylation analysis, cBioPortal provided mappings between the genomic locus of methylation and genes. The gene was assigned to one of the nearby methylation loci when a correlation of the gene and methylation loci was strongest.

**Figure 6:**
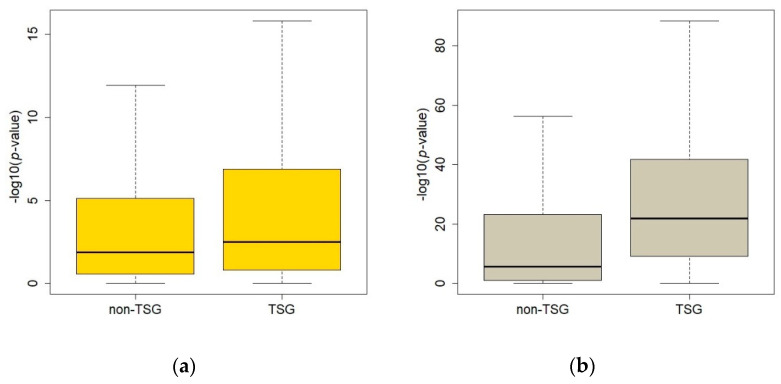
Comparison of *p*-values of the multi-omics analysis between tumor suppressor genes (TSGs) and non-TSGs. (**a**) Results of regression analysis between copy number alterations (CNAs) and methylation; (**b**) results of CNA-gene expression analysis. The difference is more obvious in the comparison of the results from the copy number and gene expression analysis. TSG: tumor suppressor genes.

CNAs and methylations of 15,027 genes were used in the CNA-methylation analyses. The TCGA breast cancer multi-omics dataset included 551 concurrent samples in methylation and CNA data. After multiple testing correction, 3528 CNA-methylation pairs were significant (Bonferroni’s adjusted *p*-value < 3.33 × 10^−6^). There were 137 TSGs in the significant result set, and 330 TSGs in the nonsignificant result set. Analysis of the Fisher’s exact test results revealed that the TSGs were underrepresented in the significant CNA-methylation pairs (odds ratio = 0.73, *p*-value = 3.23 × 10^−3^). When the *p*-values of the linear model between methylations and CNAs were compared between the TSGs (*n* = 467) and non-TSGs in the downregulated genes, the result was highly significant (Figure 6, Wilcox rank-sum test *p*-value = 2.75 × 10^–4^). 

In the CNA-expression analysis, 1068 samples had 18,090 genes in both the CNA and expression data. After regression analysis, there were 9272 significant CNA-expression pairs (Bonferroni’s adjusted *p*-value = 2.76 × 10^−6^). A total of 405 TSGs were included in the significant results, which is a highly significant overrepresentation of TSGs with Fisher’s exact test (odds ratio = 4.64, *p*-value = 2.10 × 10^−48^). The average *p*-value of linear models of CNAs and gene expressions also showed a very significant difference between TSGs (*n* = 491) and non-TSGs of the 7094 downregulated genes (Figure 6, Wilcox rank-sum test *p*-value = 1.62 × 10^−48^). 

### 2.4. Prediction of TSGs Using Results of DCG and Multi-Omics Data Analysis 

Tong et al. predicted TSGs using 75 multiple genomic features, such as sequence characteristics and epigenetic profile [31]. In the same input data, TSGs were predicted using the Neyman–Pearson classification (NPC) method described in an earlier study (Supplementary Methods) [37]. In the classification analysis, TSGs from the COSMIC database were used, because the current TSG indications were derived from the 75-feature dataset. Therefore, the TSG label is dependent on the input data used in the study. To compare the models’ prediction performance from the previous and current multi-omics data in an unbiased manner, we used the TSG labels from the COSMIC database. We applied fivefold cross-validation when estimating the performance measures. The area under the curve (AUC), prediction accuracy (PA), and sensitivity (Sen) were measured to compare model performances.

In the prediction of TSGs in breast cancer, functional genomics data were used as input data. For this purpose, statistics that were obtained from the DCG and multi-omics data analysis were treated as input data. The results of DCGs and the multi-omics data were found to be greater in TSGs than those of DCGs having no TSGs; therefore, it is possible that the DCGs results are predictors for TSGs. The *p*-values of the DCG meta-analyses were used as the input data for this analysis and a prediction model was built using the NPC method [37], which was designed to build classification models that minimize type II errors under the prespecified type 1 error rate. These results are relevant to the discovery of novel findings without sacrificing the false positive rate. The *p*-values from the DCG analyses were transformed with f(*x*) = −log_10_(*x*), and the transformed values were averaged for each gene. The averaged values were used as input data for the TSG prediction model. Of the 7318 downregulated genes, 25 had no DCGs; therefore, missing values were assigned to these genes. We also used the results of the multi-omics data analysis in the prediction analysis because they showed significant difference between TSGs and non-TSGs (Figure 4 and Figure 6). Instead of *p*-values from the testing of the fitted linear models, *t*-statistics were used to include the direction of coefficients in the prediction model. The DCG *p*-values and *t*-statistics of the multi-omics data were used simultaneously to construct a prediction model for TSGs. The platforms providing the data used in the current analysis did not include some genes; hence, some values were missing when the three statistics were used as input data for building a prediction model for TSGs. The *k*-nearest neighbor imputation method was applied to estimate missing statistics, and the prediction model was built using the same classification method [38].

Of the 7318 genes, 7290 common genes between the previous 75-feature dataset and the 7318 downregulated genes were used for the prediction analysis. When using the previous 75-feature dataset, PA was 0.95 ± 0.02, with eight different classification algorithms, and the multi-omics data showed equivalent performance (0.95 ± 0.02). However, the AUC was higher in the multi-omics data (0.54 ± 0.04) than in the 75-feature dataset (0.53 ± 0.03). The sensitivity also showed the same performance (0.03 ± 0.02) in both datasets (Supplementary Table S4).

### 2.5. Identification of Molecular Mechanism of TSGs Using Functional Enrichment Analysis

Functional enrichment analysis was applied to discover the molecular mechanism associated with TSGs. Fisher’s exact test was used in this analysis, and the alternative hypothesis was set to overrepresentation of genes of the corresponding GO terms (see Methods). The DCG analysis results show that hundreds of genes were differentially coexpressed for each gene on average. Therefore, for each TSG, multiple genes were coexpressed differentially. The list of coexpressed genes was used as an input for the enrichment analysis using Fisher’s exact test, which yielded a 508 × 7856 *p*-value matrix. Using Bonferroni’s multiple testing correction (adjusted *p*-value = 0.05/(508 × 7856) = 1.25 × 10^−8^), there were 3790 significant TSG-GOBP pairs in the result of DCG analysis (Table 2 and Supplementary Table S5). The most significantly enriched term in the obtained results was “mRNA processing” (GO:0006397), and the input genes were obtained from DCGs with CSMD1, which has a tumor-suppressive function [39]. The RNA processing related to GO terms, such as mRNA metabolic process (GO:0016071), RNA splicing (GO:0008380), and regulation of RNA splicing (GO:0043484), were found to be enriched significantly in several TSGs (see Supplementary Table S5). The biological adhesion and related GO terms such as “cell matrix adhesion” (GO:0007160), “cell migration” (GO:0016477), and “cell substrate adhesion” (GO:0031589) were also enriched significantly in many TSGs. In addition, the GO terms related to the development of vessels such as “blood vessel morphogenesis” (GO:0048514), “vasculature development” (GO:0001944), and “circulatory system development” (GO:0072359) were found to be significant. As expected, TSGs were involved in regulation of cell cycle machinery, and the cell cycle GO term was significant in the enrichment analysis in several TSGs (Supplementary Table S5).

**Figure 7:**
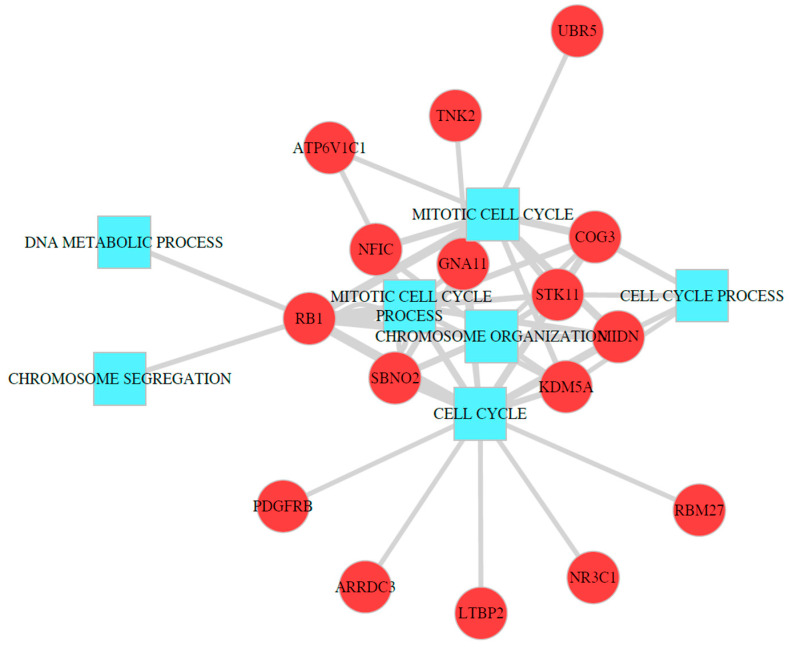
Results of meta-analysis of gene ontology enrichment analysis. The top 50 significant results are presented in a network of tumor suppressor gene (TSG) and gene ontology (GO) terms. Square nodes indicate GO terms, and the circular nodes represents corresponding TSGs. The width of edges is −log(*p*-value), and wider edges indicate more significant results.

Transcriptional regulations are a key step for molecular biologic processes; therefore, transcriptomics data provide invaluable information about the regulatory mechanism of TSGs. However, TSGs are closely related to sequence and epigenetic changes. Therefore, the identification of CNAs that affect methylations or gene expressions is likely to provide information about the regulatory mechanism of TSGs. Transregulations of the CNAs of TSGs were analyzed to examine the molecular mechanism of TSGs in cancer tissues. The main assumption of this analysis was that copy number changes of TSGs affect the methylation or gene expression of multiple genes that are related to specific biological functions. Moreover, if the results of CNA methylation and CNA expression analyses are integrated, more statistical power could be expected. Therefore, for the CNAs of each TSG, methylations and gene expressions that had a significant linear relationship with the CNAs were used in the GO enrichment analysis, and enrichment *p*-values were integrated using the modified Brown method. In the copy number data for TCGA breast cancer, 508 TSGs of the downregulated genes were included, and the association of methylations and gene expressions were tested using a linear regression model. In the results, many genes showed significant results in the regression analysis according to the CNA of the TSGs. The significant genes were used for the GO enrichment analysis. Moreover, after including the enrichment analysis results for the DCGs, the *p*-values from the enrichment analysis were integrated for each GOBP term (see the Supplementary Methods). The same data were redundantly used in the multi-omics data and DCG analysis; thus, the *p*-values were integrated using the modified Brown method. The results of the integrative analysis were consistent with the current biological knowledge of the role of TSGs. The RB1 and “cell cycle” (GO:0007049) terms showed the most significant result (Figure 7 and Supplementary Table S6). Among the highly significant results, many genes showed significant enrichment with the “cell cycle” and related terms (see Supplementary Table S6). In addition to the “cell cycle” term, “chromosome organization” (GO:0051276) and related terms including “chromosome segregation” (GO:0057059), “nuclear chromosome organization” (GO:0000228) and “regulation of chromosome organization” (GO:0033044) were significant. As found in the results of the DCG analysis, “biological adhesion”, “RNA processing”, and related terms were included in the results.

## 3. Discussion

In this research, regulatory mechanisms of TSGs were identified through meta-analyses of RNA sequencing and multi-omics data for breast cancer. The results revealed that TSGs have stronger regulatory relationships with other genes than non-TSGs, and that ribosome-related processes are significantly enriched in the transcriptional regulation of TSGs. Using the results of DCG and multi-omics data analyses, the enrichment tests revealed the various molecular processes of TSGs.

In this study, TSGs were selected from a previous study [31]. In the research, the TSGs were predicted using the genetic and epigenetic features that were obtained from heterogeneous sources, including the COSMIC database [40] and ENCODE project [41]. The predicted TSGs were used to obtain a reliable list of TSGs determined using independent sources. Moreover, for the identification of breast cancer-specific TSGs, the intersections of the TSGs and downregulated genes were used as tentative TSGs specific to breast cancer. Of the 508 TSGs, only 21% (*n* = 107) were listed in the COSMIC database, and the rest of the TSGs were considered novel. In the previous report, TSGs tended to show low fold changes between normal and cancer tissues [34]. Therefore, instead of using highly significant results, all downregulated genes were used in the selection of TSGs specific to breast cancer.

The degree of differential coexpressions between TSG and non-TSG groups changed substantially, which is consistent with earlier studies. Anglani et al. reported that coexpression patterns in normal and cancer tissues are highly different, which results in the loss of connectivity in the topology of the coexpression network of cancer driver genes [42]. This result implies that there are significant differential coexpressions with important genes for malignant transformation, because the loss of connectivity indicates a substantial change in coexpressions between normal and cancer tissues [42]. The same tendency was identified in another study that reported reduced network complexity in cancer transcriptomics data compared with normal tissues. As in Anglani’s study, the reduced complexity indicated the loss of coexpression connections in cancer tissues [34]. The current analysis showed that all the coexpressions of the downregulated genes have negative values in breast cancers, while the values are mostly positive in normal breasts. The opposite sign of the coexpressions between normal and cancer tissues indicates there are substantial changes of connectivity of coexpression network, as in the previous studies. Moreover, *p*-values of the DCG analysis showed significant different between DCG with the TSGs and non-TSGs, which indicates the changes of the connectivity are greater in TSGs-connected genes. Given these, differential coexpressions between normal breasts and breast cancers provide an information for discriminating TSGs and non-TSGs. 

The performance of predicting TSGs was substantial in this analysis, especially compared with the results of the other studies. This might result from the selection of DEGs and DCGs. While the prediction of TSGs in the previous study used genomic and epigenomic features, functional genomics data were used in this analysis. Moreover, DEGs were computed using a meta-analysis, which has more power in the detection of DEGs than analysis of single studies. Therefore, estimation of DEGs might be a filtering process for the detection of TSGs. In addition, DCG and the multi-omics data analysis provided substantial information to the prediction of TSGs. 

One of the most notable findings in this research is that the ribosome pathway was found to be more frequently associated with the gene regulation of TSGs than non-TSGs in the downregulated genes. Ribosome is a critical cellular component for protein synthesis, and dysregulation of ribosome biogenesis was found to be associated with progression of breast cancer cells [43,44,45]. Since the ribosomal RNA or protein modifications affect cancer development [46,47], it is possible that differential regulations of ribosomal genes might influence cancer cell development via TSGs. Considering this, the potential of the ribosomal pathway should be investigated for the discovery of strategies to prevent or treat cancer through the control of the TSGs in breast cancer. 

The DCGs of the TSGs showed the enrichment of specific biological processes. For example, ‘mRNA processing’ or related GO terms were strongly enriched with TGSs. The related GO terms included mRNA metabolic processing, RNA processing, and RNA splicing. These GO terms were top-ranked according to the *p*-values of enrichment analysis, and the results seem to be consistent with the enrichment of the ribosome-related biological processes, because RNA processing and ribosomes are coupled in the protein expression of genes. In the results, angiogenesis-related GO terms such as “circulatory system development”, “vascular development”, and “blood vessel morphogenesis” were included, and this finding is consistent with previous findings. For example, FOXP3 downregulates VEGF, leading to inhibition of angiogenesis in breast cancer [48]. G-protein gamma subunit 2 (GNG2), which is involved in angiogenesis, was recently found to be a TSG [49]. In addition to RNA processing and angiogenesis, metastasis-related GO terms such as “biological adhesion”, “regulation of cellular component”, and “cell migration” were highly enriched. There is a great deal of evidence that TGSs are associated with metastasis of cancer cells [50,51,52,53]. Well-known TSGs, such as PTEN and Rho GTPases, play major roles in breast cancer metastasis [50,53]. These observations indicate that the significantly enriched GO terms for the DCGs with TSGs show clues about the molecular mechanism of genetic regulation of TSGs, which will provide invaluable information for studying the individual genes involved in the process. 

The molecular mechanisms of TSG were also clearly defined in the integrative analysis of multi-omics data. The results showed that a TSG had significant links between multiple GO BP terms, and the terms were very consistent with previous findings on TSGs. The significantly enriched GO terms were related to cell cycle, genome stability, mRNA metabolism, cell adhesion, and immune response, which were already identified as core mechanisms of TSGs in the oncogenic transformation [13]. Well-known TSGs such as RB1, APC, and P63 showed significant results with the GO terms. Moreover, HDAC2, which is involved in epigenetic changes, showed significant results with the GO terms. Interestingly, while cell-cycle- or genome-stability-related GO terms were frequently ranked as highly significant results in the other TSGs, cell adhesion GO terms that are associated with metastatic activity were highly ranked in the result of HDAC2 enrichment. This result was consistent with previous reports on HDAC2 [54,55], and indicated that the result of this analysis provides information about the specific molecular mechanisms of TSGs. 

In this study, novel findings regarding TSGs in breast cancer were identified. For example, in the DCE analysis, the MRAS and ITPK1 gene pair had the most significant *p*-value. MRAS is a member of the RAS family, which is a family of well-known oncogenes that are involved in the development of various diseases [56]. ITPK1 is an enzyme that produces inositol 3,4,5,6-tetrakisphosphate, which is an inhibitor of calcium-activated chloride channels [57]. The findings of the present study indicate that increased expression of MRAS is linked to decreased expression of ITPK1 in breast cancer, which might lead to underproduction of inositol phosphate 6 (IP_6_) [58]. IP_6_ is known to have growth inhibitory effects in breast cancers, and it enhances the antiproliferative effect of chemotherapeutic agents, such as adriamycin and tamoxifen [59]. Considering this information, the possible oncogenic effect of MRAS can be expected to be suppressed by positive coexpression of ITPK1 and reversed in breast cancer. This indicates that ITPK1 is a possible TSG. Another example is the significant enrichment of RNA processing-related GO terms in the list of genes differentially coexpressed with GATA1 (Table 2). GATA1 showed high significance in the enrichment analysis of the results from the DCE analysis regarding TSGs and the other downregulated genes. The significant GO terms included “RNA processing”, “mRNA metabolic processing”, and “RNA splicing” (Table 2 and Table S5). While GATA1 is known to be involved in the epithelial–mesenchymal transition process [60], no RNA processing genes are known to be associated with GATA1. The splicing factors are known to have tumor suppressive functions [61]. Therefore, it is possible that GATA1 and the genes involved in RNA processing, including genes for RNA splicing, might have a cooperative tumor suppressive function. In the integrative analysis of multi-omics data, GO terms that were relevant to the mechanism of TSGs were identified, which indicates that the biological processes of GO terms were manifested by the orchestration of multiple molecular steps, including methylation and gene expression. Moreover, some genes have such molecular mechanisms, but which have not been reported previously. The COG3 gene was significantly linked to the “cell cycle” GO term, and this finding appeared to be novel because COG3 is known to be involved in the tethering of transport vesicles [62]. However, RhoBTB, which is one of the TSGs, has been reported to regulate the integrity of the Golgi complex [50]. Because COG3 was one of the predicted TSGs, it might have functions in transport and regulation of genes involved in cell cycle machinery.

## 4. Materials and Methods

### 4.1. Breast Cancer Multi-Omics Data

The RNA sequencing data for normal breast tissues were downloaded from the GTEx project website [63]. The GTEx project was designed to build a comprehensive public resource of providing data and information about tissue-specific gene expressions and the relationship between genetic variations and gene expressions [64]. Gene read counts of the GTEx analysis V8 were downloaded, and gene expression counts of normal breast tissues were used.

The TCGA breast cancer dataset was downloaded from the cBioPortal website [65]. The dataset holds genome-wide CNAs, gene expressions methylations, and protein expressions information. The CNA data had five levels of alterations, including −2, −1, 0, 1, and 2. In the TCGA project, the CNAs were estimated using the Affymetrix 6.0 SNP platform and the GISTIC algorithm [66]. The estimated CNAs were provided in the cBioPortal, and the data were used in this analysis. RNA sequencing data contained counts of gene expressions estimated by the RNA-Seq by expectation-maximization method [67], and underwent batch-adjustment [30]. Methylation profiles were obtained using the Infinium HumanMethylation450 BeadChip (Illumina^®^, San Diego, CA, USA), and among the beta values of the array probes of genes, probes having the most anti-correlations with the corresponding gene expressions were used as degree of methylation in the genes. All data had gene symbols, which were used for integrative analysis with the functional genomics datasets from different platforms.

For the meta-analysis of breast cancer gene expression data, RNA-seq data that had a large number of samples, including GSE96058 and GSE81538, were collected from the Gene Expression Omnibus database [68]. 

### 4.2. Identification of Differentially Expressed Genes and Co-Expressed Genes

Various methods based on the *t*-test or linear models were used in the analysis of DEGs [69,70]. In this analysis; however, we applied a rank-based statistics approach to remove the batch effect of RNA-seq data. Genomic data are prone to batch effects that show different statistical distribution between different batches of genomic data, which cannot be fully resolved with normalization methods [71]. Moreover, it is possible that raw data are unavailable for the meta-analysis. In this analysis, gene expression ranks were used in the DEG analysis. First, a gene expression vector of a sample was sorted in an ascending order, and higher expression values were transformed into higher ranks. We then compared the gene expression ranks between the normal and breast cancer expression data. In the comparison, the Wilcoxon rank-sum test was applied to deal with the rank-transformed expression values. The batch effect was applied to all genes of samples in a batch equivalently; therefore, the ranks between the gene expression values in a sample can be preserved. However, the batch effect can be circumvented with the comparison of ranks between normal and cancer samples.

The identification of DCEs between genes was performed with the determination of PCC for the gene pairs in normal and cancer samples separately. The differences between PCC results were determined with a statistical test:

(1) $$PCC=\frac{\sum \left(x_{1}-{\bar{x}}_{1})(x_{2}-{\bar{x}}_{2}\right)}{\sqrt{\sum {\left(x_{1}-{\bar{x}}_{1}\right)}^{2}}\sqrt{\sum {\left(x_{2}-{\bar{x}}_{2}\right)}^{2}}}$$

In Equation (1), *x*_1_ and *x*_2_ indicate gene expression vectors. In the determination of DCE, a hard thresholding approach can be applied to increase confidence of the DCE analysis, which consists of the determination of the statistical significance of the PCC results under different conditions, which include normal breasts and breast cancer in this analysis. The PCC was transformed into *t*-test statistics using Equation (2):

(2) $$t=\frac{PCC\sqrt{n-2}}{\sqrt{1-PCC^{2}}}$$

where *n* indicates the number of samples in a condition. The *p*-value of the test was determined by the *t* distribution with *n*—2 degrees of freedom. The difference of the PCCs between conditions was tested with standard normal distribution. Test statistics were obtained from PCCs for two conditions with Equation (3):

(3) $$dz=\frac{z_{1}-z_{2}}{\sqrt{1/(n_{1}-3)+1/(n_{2}-3)}}$$

In the above equation, *z* was determined by 0.5log((1 + *PCC*)/(1 − *PCC*)), where *n* indicates the number of samples in each condition as indicated by subscripts.

### 4.3. Meta-Analysis Using p-Value Combination

Fisher’s *p*-value combination was applied when performing the meta-analysis of the results from the DEG and DCE analyses. The method uses the summation of the –log(*p*-value) from each study, and the statistics were applied to the Chi-square distribution:

(4) $$\chi_{2k}^{2}\sim -2\sum_{i=1}^{k}log(P_{i})$$

where *k* indicates the degree of freedom, which is equivalent to the number of studies included in the meta-analysis. In the original form of the Fisher’s *p*-value combination method, *p*-values should be independent. In this analysis, however, the *p*-values are correlated between studies because the same control data for normal breasts were used, leading to inflation of the test statistics. We applied the empirical Brown method to resolve this issue. The empirical Brown method uses a rescaled Chi-square distribution in the application of the Fisher’s method to dependent *p*-values [35]:

(5) $$\psi \sim c\chi_{2f}^{2}$$

To compute *c* and *f*, the expected value and variance of Ψ is required. In the empirical Brown method, they are estimated using numerical integration in Equation (6):

(6) $$E\left[\psi \right]=2k,\mathrm{var}\left[\psi \right]=4k+2\sum_{i<j}\mathrm{cov}(-\log P_{i},-\log P_{j})$$

In the empirical Brown method, the covariance of the Chi-square statistics is inferred by Equation (7):

(7) $$\mathrm{var}\left[\psi \right]=4k+2\sum_{i<j}\mathrm{cov}({\vec{w}}_{i},{\vec{w}}_{j})$$

where the $\vec{w}$ is estimated by $-2\log(1-F({\vec{x}}_{i}))$, and $F(\vec{x})$ is the right-sided empirical cumulative distribution function computed from the sample vector of $\vec{x}$.

### 4.4. Functional Enrichment Analysis with Gene Ontology and Pathway Information 

To reveal the molecular mechanisms of DCGs and multi-omics data analysis, a functional enrichment analysis was performed using Fisher’s exact test. When using a functional gene set, the following equation was used.

(8) $$p=\frac{\left(a+b\right)!\left(c+d\right)!\left(a+c\right)!\left(b+d\right)!}{\left(N!a!b!c!d!\right)}$$

In Equation (8), *a* is the number of genes that are included in both the DCGs and gene sets, *b* is the number of overlapped genes between DCGs and genes that are not included in the gene sets, *c* indicates the number of common genes between non-DCGs of the reference genes and the gene sets, and *d* is the number of overlapping genes of the non-DCGs and genes outside the reference gene sets.

The gene sets included GOBP terms and KEGG pathway information. The gene symbols for each gene set were downloaded from the Gene Set Enrichment Analysis website [72].

### 4.5. Multi-Omics Data Analysis with Linear Model

A simple linear regression was applied to identify the impact of CNAs on methylations and gene expressions. Using a regression model of Y ~ CNA, where Y is the methylation or gene expression values, the relationship between two elements were tested. The significant result of the linear model indicates that the methylation or gene expression levels change according to the change in copy number status and the changes are not random events. For a single CNA, the analysis used multiple methylations or gene expressions as predictor variables and used Bonferroni’s multiple testing correction for the determination of statistical significance.

## 5. Conclusions

In this study, molecular characterization of the regulatory mechanisms of TSGs and other genes was performed via a meta-analysis of transcriptomics data and an integrative analysis of breast cancer multi-omics data. To my knowledge, the integration of the results of DCE and multi-omics data analyses using a summation of the *p*-values from GO enrichment analysis is a novel approach that has never been applied elsewhere. Although some of the identified regulatory mechanisms of TSGs are already known, the determination of links between TSGs and the mechanisms is a unique feature of this analysis. The present study findings would provide invaluable information for the development of preventive or therapeutic strategies for breast cancer.

## Figures and Tables

**Table 1:** Top 10 significant results of DCG analysis. The degree of coexpression is represented with correlation coefficients.

Gene1	Gene2	GTEx	GSE90538	GSE81555	TCGA	*p*-Value
MRAS	ITPK1	0.84	–0.38	–0.31	–0.10	1.09 × 10^–298^
ESYT3	IFT140	0.80	–0.33	–0.39	–0.35	1.80 × 10^–290^
CDK5RAP3	ZNF667	0.87	–0.18	–0.11	–0.12	7.42 × 10^–287^
PLA2R1	PDE7A	0.86	–0.20	–0.21	–0.13	8.47 × 10^–287^
ESYT3	CMYA5	0.84	–0.27	–0.34	–0.10	1.06 × 10^–283^
PEX19	LDHB	0.83	–0.39	–0.30	–0.05	7.00 × 10^–282^
ACACB	ABCD1	0.84	–0.32	–0.19	–0.10	4.63 × 10^–278^
TTLL4	JMJD7	0.80	–0.33	–0.47	–0.22	3.49 × 10^–276^
ESYT3	DTX3	0.83	–0.25	–0.32	–0.23	3.40 × 10^–275^
SFT2D2	RAD50	0.83	–0.28	–0.32	–0.12	3.36 × 10^–272^

GTEx: Gene Tissue Expression, TCGA: The Cancer Genome Anatomy.

**Table 2:** Significant results of functional enrichment analysis. Please note that the same gene is significantly linked to related gene ontologies.

Gene Name	GO BP	*p*-Value
CSMD1	mRNA PROCESSING	8.96 × 10^–36^
GATA1	mRNA METABOLIC PROCESS	6.38 × 10^–34^
CSMD1	mRNA METABOLIC PROCESS	1.07 × 10^–33^
GATA1	mRNA PROCESSING	6.33 × 10^–32^
BNC2	mRNA METABOLIC PROCESS	1.45 × 10^–31^
BNC2	mRNA PROCESSING	2.04 × 10^–30^
BNC2	RNA PROCESSING	6.50 × 10^–29^
GATA1	RNA PROCESSING	2.40 × 10^–28^
BNC2	RNA SPLICING	9.30 × 10^–28^
PTPRD	mRNA METABOLIC PROCESS	1.96 × 10^–27^

Go: gene ontology, BP: biological process.

## Supplementary Materials

The following supporting information can be downloaded at: https://www.mdpi.com/article/10.3390/ijms23179624/s1.
