# Supplementary material for: Uncovering Oncogenic Mechanisms of Tumor Suppressor Genes in Breast Cancer Multi-Omics Data
Source: Int J Mol Sci. 2022 Aug 25;23(17):9624. doi: 10.3390/ijms23179624 (PMC9455665; doi:10.3390/ijms23179624)
Supplement: Supplementary file 1 [file ijms-23-09624-s001.zip › ijms-1824056-Supplementary Methods.pdf]

Supplementary Methods

Machine learning-based prediction of tumor suppressor genes

For the prediction of TSGs using the statistics of DCGs and multi-omics data analysis, the NPC method was applied. The NPC is a paradigm that minimizes the type II error in the prediction using classification algorithms [1]. The task can be formalized as:

$$\phi_{\alpha}^{*} = \arg\min_{\phi:R_0(\alpha)\leq\alpha} R_1(\phi) \tag{1}$$

In Equation (1), φ is a classifier for class 0 and 1, and α indicates an upper bound of type 1 error. The α is determined before the classification is performed. The *R*() is a risk function for classifier φ that is a weighted sum of type 1 and type 2 error, and R_0 and R_1 are the risk function for class 0 and class 1, respectively. The core concept of the NPC is the violation rate that is a probability derived from Equation (2).

$$P[R_0\left(\hat{\phi}_k\right) > \alpha] \leq \sum_{j=k}^{n} \binom{n}{j} (1-\alpha)^j \alpha^{n-j} \tag{2}$$

where the *k* indicates the rank of the score produced by a classification model, and *n* is the number of class 0 samples. Consequently, the NPC determines a threshold of classifier scores that minimize type 2 error under a predefined type 1 error rate. The NPC *R* package was used for the NPC analysis in the prediction of the TSGs.

Test for enrichment of differential coexpressions with tumor suppressor genes

In the differential coexpression (DCG) analysis, a single gene has multiple genes that are coexpressed differentially. In the genes, tumor suppressor genes (TSGs) or non-

TSGs can be included. To identify the gene that have DCG relationship with TSGs more frequently, Fisher's exact test was applied. As shown in Fig. S1, for each down-regulated genes, the number of DCGs in TSGs and non-TSGs were compared. And, Bonferroni's multiple testing correction was applied.

Fig. S1. Testing of enrichment of tumor suppressor genes (TSGs) in the result of differential coexpression (DCG) analysis.

		TSG				non-TSG				
**Gene**	**G1**	**G2**	**G3**	**G4**	**G5**	**G6**	**G7**	**G8**	**G9**	**G10**
DCG result		1	1	1	0	1	0	0	0	0

	**TSG**	**non-TSG**
DCG (+)	3	1
DCG (-)	1	4

For a gene (G1), results of the DCG analysis with resting genes (G2 ~ G10) were summarized into a vector of 0 and 1, which indicates significance of DCG (significant result = 1). Fisher's exact test was applied to detect whether the number of significant DCG results were enriched in TSGs or not.

Integrative analysis of differential coexpression, multi-omics and gene set enrichment analysis.

In the DCG and multi-omics data analysis, many genes can be found to be significant

for a gene. The multiple genes were used as an input gene list in the gene set enrichment analysis. After the enrichment analysis, p-values of gene ontology terms that were used in the analysis were obtained. Consequently, three p-value vectors were available from the DCG, CNA-methylation, and CAN-expression multi-omics data analysis. These p-value vectors were integrated using the modified Brown method that integrates a covariance structure between the p-values (Fig S2).

Fig. S2. Integrative analysis of differential coexpression, multi-omics data, and gene set enrichment analysis.

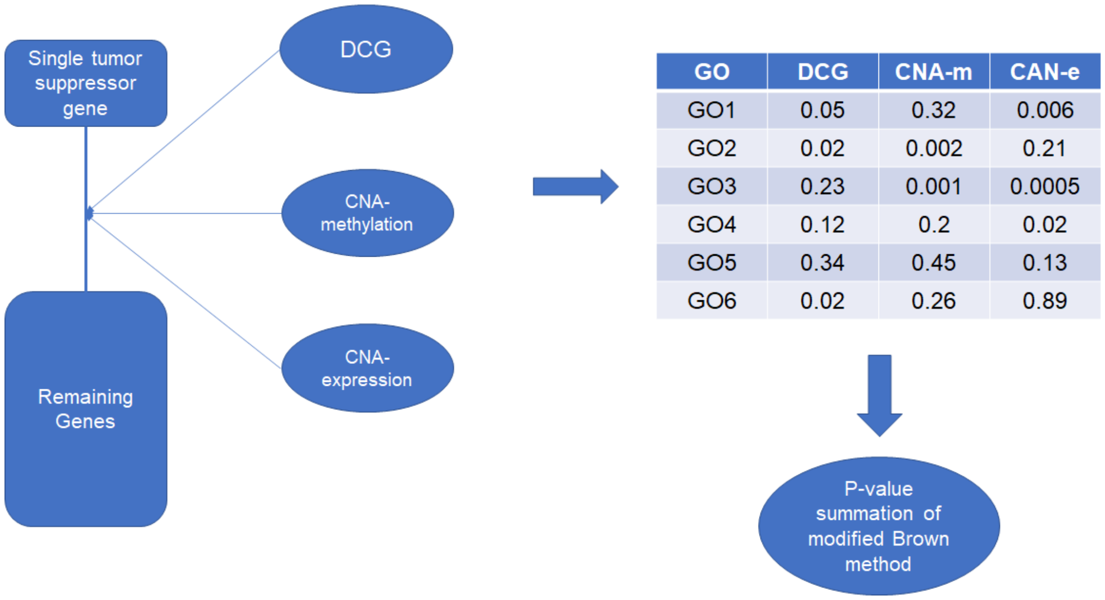

The matrix contains p-values from the three different analyses. DCG: differential coexpression, CNA: copy number alterations. CNA-m: CNA-methylation analysis, CNA-e: CNA-expression analysis.

Reference

1. Tong X, Feng Y, Li JJ. Neyman-Pearson classification algorithms and NP receiver operating characteristics. Sci Adv. 2018 Feb 2;4(2)
